# Prevalence of Parathyroid Carcinoma and Atypical Parathyroid Neoplasms in 153 Patients With Multiple Endocrine Neoplasia Type 1: Case Series and Literature Review

**DOI:** 10.3389/fendo.2020.557050

**Published:** 2020-09-30

**Authors:** An Song, Yi Yang, Shuzhong Liu, Min Nie, Yan Jiang, Mei Li, Weibo Xia, Ou Wang, Xiaoping Xing

**Affiliations:** ^1^Key Laboratory of Endocrinology, Ministry of Health, Department of Endocrinology, Peking Union Medical College, Peking Union Medical College Hospital, Chinese Academy of Medical Sciences, Beijing, China; ^2^Department of Orthopaedic Surgery, Peking Union Medical College Hospital, Peking Union Medical College and Chinese Academy of Medical Sciences, Beijing, China

**Keywords:** multiple endocrine neoplasia type 1, parathyroid carcinoma, atypical parathyroid neoplasms, prevalence, Chinese population

## Abstract

**Purpose:** The occurrence of parathyroid carcinoma (PC) and atypical parathyroid neoplasm (APN) in multiple endocrine neoplasia type 1 (MEN1) is rare. The present paper reports the cases of 3 MEN1-PC/APN patients at our center and discusses the prevalence in a Chinese MEN1 cohort.

**Methods:** This report is a retrospective analysis of 153 MEN1-associated primary hyperparathyroidism (MEN1-HPT) patients at our center, which included 3 MEN1-associated PC/APN (MEN1-PC/APN) patients. The clinical manifestations, biochemical indices, pathological findings, and therapy have been summarized along with the report of the genetic testing of the 3 patients.

**Results:** Of the 153 MEN1-HPT patients, 1 (0.7%) was histopathologically diagnosed with PC and 2 (1.3%) with APN. Three heterozygous mutations were identified in the 3 MEN1-PC/APN patients (c.917 T > G, c.431T > C, and c.549 G > C). The cumulative findings of 3 cases with 18 previously reported MEN1-PC/APN cases revealed that the mean serum calcium (Ca) level was 3.15 ± 0.44 mmol/L and the median parathyroid hormone (PTH) level was 327 pg/mL (214.1, 673.1), both of which were significantly higher as compared to the respective levels in non-PC/APN MEN1 patients at our center [Ca: 2.78 mmol/L [2.61, 2.88], PTH: 185.5 pg/mL [108.3, 297.0]; *P* = 0.0003, 0.0034, respectively].

**Conclusion:** MEN 1-PC/APN is a rare disease, with a prevalence of only 2.0% among the MEN1-HPT cohort at our center. The affected patients recorded higher serum Ca level and PTH levels than those with MEN1-associated benign tumors. However, the diagnosis of MEN1-PC/APN is based upon pathology most of the times.

## Introduction

Multiple endocrine neoplasia type 1 (MEN1) is an autosomal dominant hereditary syndrome that is characterized by the presence of endocrine tumors affecting the parathyroid, pancreas, and pituitary. In addition, some of these patients also develop adrenal adenoma (AA), carcinoid tumors (CT), angiofibroma, lipomas, collagenoma, and meningiomas (1). MEN1 is diagnosed based on the clinical or genetic criteria, and it has a prevalence of ~2–20 cases in 100,000 (1). Primary hyperparathyroidism (PHPT) is the most common manifestation of this syndrome, recorded in >95% of cases, with ~100% penetrance at the age of 50 years (2). The most frequent pathological type of MEN1-associated PHPT (MEN1-HPT) is parathyroid hyperplasia, which often involves multiple glands simultaneously (3, 4).

In contrast, in sync with the tendency of malignant pathology, the occurrence of parathyroid carcinoma (PC) and atypical parathyroid neoplasm (APN) as the causes of PHPT in MEN1 is a rare possibility. Until date, only 17 MEN1-associated PC (MEN1-PC) cases and only 1 MEN1-associated APN (MEN1-APN) case have been reported in the literature, accounting for a prevalence rate of 0.28–1% among MEN1 patients (5, 6). However, these literature are limited to the Caucasian population, and the corresponding data relevant to the Chinese or other Asian population remains lacking.

In this report, we have summarized the clinical manifestations, treatments, and genetic backgrounds of 3 MEN1-PC/APN patients admitted to our center as well as have reported the prevalence of MEN1-PC/APN in a Chinese cohort of MEN1 patients.

## Materials and Methods

### Subjects

Between 1999 and 2019, 153 MEN1-HPT patients belonging to 148 families were diagnosed at the Department of Endocrinology, The Peking Union Medical College Hospital (PUMCH), Beijing. Among them, 112 patients underwent surgical treatment for MEN1-HPT, of which 3 were pathologically diagnosed with PC or APN.

### Diagnosis

The diagnosis of MEN1 was established by one of the 3 criteria, namely (2, 4): (i) Clinical criteria: the occurrence of two or more major MEN1-associated endocrine tumors [i.e., parathyroid tumor, pancreatic endocrine tumor (PET), and pituitary adenoma [PIT]]; (ii) Genetic criteria: the identification of a germline MEN1 mutation, who might be asymptomatic with no biochemical or radiological manifestations of MEN1; and (iii) For the first-degree relatives of MEN1-patients: The occurrence of one MEN1-associated tumor.

The diagnostic criteria for PC relied on finding lesions with vascular invasion, perineural invasion, capsular penetration, and/or documented metastases (7). The APN was diagnosed for parathyroid tumors partially sharing some of the atypical features in PC, but not meeting all the histological criteria of PC (6).

### Clinical Investigation

The clinical data were retrospectively collected, including general characteristics, clinical manifestations (e.g., bone involvement, urinary system damage, gastrointestinal symptoms, and hypercalcemia crisis), treatment strategies, pathological features, and follow-up (postoperative recurrence and metastasis). Bone involvement included bone pain, pathological fracture, and the X-ray features of PHPT characterized by bone resorption (such as subperiosteal absorption and osteitis fibrosa cystica). Bone mineral density (BMD) was measured by dual-energy X-ray absorptiometry (DXA; GE-Lunar, USA). Urolithiasis or renal calcification was assessed via ultrasound application.

The hypercalcemia crisis was defined as a serum total calcium (Ca) level of ≥3.5 mmol/L, and it is usually associated with development of acute signs and symptoms of hypercalcemia (8). The definition of recurrence was presentation of hypercalcemia after a disease-free period of at least 6 months after parathyroidectomy (9).

### Laboratory Examinations

Biochemical indices including serum Ca, phosphorus (P), alkaline phosphatase (ALP), and 24-h urine Ca (24-hUCa) were measured with the Beckman Automatic Biochemical Analyzer (AU5800; Beckman Coulter). Ionized-Ca (iCa) levels were measured with a blood-gas analyzer radiometer (ABL800 FLEX; Denmark). The serum parathyroid hormone (PTH) level was measured via chemiluminescence (ADVIA Centaur; Siemens, Germany). Serum 25-dihydroxyvitamin D (25OHD) value was measured using an electrochemiluminescence immunoassay (e601; Roche Cobas, Germany). Normal reference ranges for indices: serum Ca: 2.13–2.70 mmol/L. serum iCa: 1.08–1.28 mmol/L, serum P: 0.81–1.45 mmol/L, ALP: 30–120 U/L, PTH: 13–65 pg/mL, 25OHD: 30–50 ng/mL, 24hUCa: <7.5 mmol.

### DNA Isolation and Gene-Mutation Analysis

Genomic DNA was extracted from the peripheral blood lymphocytes using the QIAamp Blood DNA Kit (Qiagen; Hilden, Germany). All coding exons and exon-intron boundaries of the *MEN1* were amplified via polymerase chain reaction (PCR), followed by Sanger sequencing which was performed as previously prescribed (10).

### Statistical Methods

Statistical analysis was conducted using the Medcalc software (Version 18.2, Ostend, Belgium). All the MEN1-HPT patients at our center, except for the 3 MEN1-PC/APN patients, were placed into the non-PC/APN group. All the 18 reported MEN1-PC/APN cases and 3 from our center were placed in the PC/APN group. All data that were normally distributed were described by mean and standard deviation, and group differences were compared using independent *t-*tests. Data that were non-normally distributed were described in median and 25th and 75th interquartile ranges (Q25, Q75), while group differences were compared using rank-sum tests. The pathological results of the patients were clearly diagnosed by 2 pathologists. For patients with multi-glandular involvement, if the pathological findings were different for different lesions, the diagnosis was based on the findings of the more malignant areas. *P* < 0.05 was considered to be statistically significant.

## Results

### Clinical Data and Genetic Screening of MEN1-PC/APN Patients at Our Center

A total of 153 patients with a clinical and/or genetic diagnosis of MEN1-HPT were identified at our center. Of these, 112 patients (73.2%) underwent surgical treatment, of whom 45 had hyperplasia (40.2%), 64 had adenomas (57.1%), 2 had APN (1.8%), and 1 had PC (0.9%).

The mean disease onset age in the non-PC/APN group (*n* = 150) was 43.0 ± 15.5 years (range: 12–77 years), with a median course of PHPT of 6 years (range: 2 weeks−35 years). This group included 87 women (58%) and 63 men (42%). The involvement of the gastrointestinal tract, urinary tract, and bone were noted in 32, 74, and 70 patients in the non-PC/APN group (21.3, 49.3, and 46.7%), respectively. Bone involvement included 29 cases with bone pain, 14 cases with pathological fracture, 12 cases with subperiosteal absorption, 5 cases with osteitis fibrosa cystica and 43 cases with osteoporosis measured by DXA. The median serum Ca level was 2.78 mmol/L (2.61, 2.88), median PTH was 185.5 pg/mL (108.3, 297.0), and median 24-h UCa was 7.68 mmol (5.09, 10.28) in this group.

Table 1 summarizes the general characteristics of the 3 MEN1-PC/APN patients. The age at which the disease onset was noted in all 3 cases was >49 years (the mean onset age of the non-PC/APN group was 43.0 ± 15.5 years) with a relatively long course of disease. All the 3 patients had other MEN1 diseases, including PET, PIT, and AA. Table 2 presents the clinical manifestations and preoperative biochemical markers of the 3 patients. All of them had nephrolithiasis, 2 had bone pain with osteoporosis, and 1 presented with gastrointestinal symptoms such as nausea and vomiting. None of the 3 patients demonstrated hypercalcemia crisis. Table 3 summarizes the details of the surgical methods, histopathological features, and postoperative follow-up of the 3 patients.

**Table 1:** General characteristics of MEN1-PC/APN cases in our center.

**Case**	**Sex**	**Age (years)**	**Course of disease (years)**	**MEN1 family history**	**First involved gland**	**Other MEN1 diseases**
P1	Female	51	27	Yes	Parathyroid	Pancreatic non-functional tumor, adrenal non-functional tumor
P2	Female	52	10	No	Pituitary	Pituitary prolactinoma
P3	Male	49	12	Suspicious	Parathyroid	Pancreatic neuroendocrine tumor, adrenocortical adenoma, pituitary microadenomas

**Table 2:** Clinical manifestations and biochemical indices of MEN1-PC/APN cases in our center.

**Case**	**Skeletal involvement**	**Urinary involvement**	**Gastrointestinal symptoms**	**Hypercalcemia crisis**	**Ca (mmol/L)**	**iCa (mmol/L)**	**P (mmol/L)**	**ALP (U/L)**	**PTH (pg/mL)**	**25OHD (ng/mL)**	**24hUCa (mmol)**	**24hUP (mmol)**
P1^*^	Yes	Yes	Yes	No	2.56	1.31	0.96	219	2189.4	25.4	4.13	1.89
P2	No	Yes	No	No	3.35	/	0.87	195	708.8	/	22.56	/
P3	Yes	Yes	No	No	2.83	1.39	0.74	113	380	6.1	7.32	19.74

Note: 1. * The patient underwent two times operations; the pathology was adenomas for the first time and atypical adenomas for the second time. The results in this table were before the second operation. The results of P2 and P3 were before the first operation. *2. Normal reference ranges for indices: Ca (serum calcium): 2.13–2.70 mmol/L. iCa (serum ionized calcium): 1.08–1.28 mmol/L. P (serum phosphorous): 0.81–1.45 mmol/L. ALP (alkaline phosphatase): 30–120 U/L. PTH (serum parathyroid hormone): 13–65 pg/mL. 25OHD (25-hydroxyvitamin D): 30–50 ng/mL. 24hUCa (24h urinary calcium): <7.5 mmol*.

**Table 3:** Treatment and follow-up of MEN1-PC/APN cases in our center.

**Case**	**Surgery**	**Pathology**	**Histopathologic features**	**Tumor diameter (cm)**	**Number of lesions**	**Postoperative**		**Follow-up (year)**	**Recurrence/ metastasis**
						**Ca (mmol/L)**	**PTH (pg/ml)**		
P1^*^	Total parathyroidectomy + auto plantation	Atypical adenoma (right superior and right inferior) Hyperplasia (left inferior)	Lobular features, fibrous bands, focal active growth and mitoses	2.5	3	2.02	120.4	1	No
P2	Left superior and right inferior parathyroidectomy	Atypical adenoma	Active growth, mitoses and thick bands	2.3	2	2.24	21	3	No
P3	Left inferior and right superior parathyroidectomy	Carcinoma	The cubical or rounded cancer cells are small, stratified in layers as a ridge, focal thick bands, capsular invasion, suspicious vascular invasion, focal oncocytic cells	/	2	2.39	/	4	Recurrence

Note: 1. * This patient underwent two times operations. The pathology was adenomas for the first time, and atypical adenomas for the second time. The results in this table are about the second operation. *2. Ca (serum calcium): 2.13–2.70 mmol/L. PTH (serum parathyroid hormone): 13–65 pg/mL*.

### Case Report

#### Case 1

**Figure 1:**
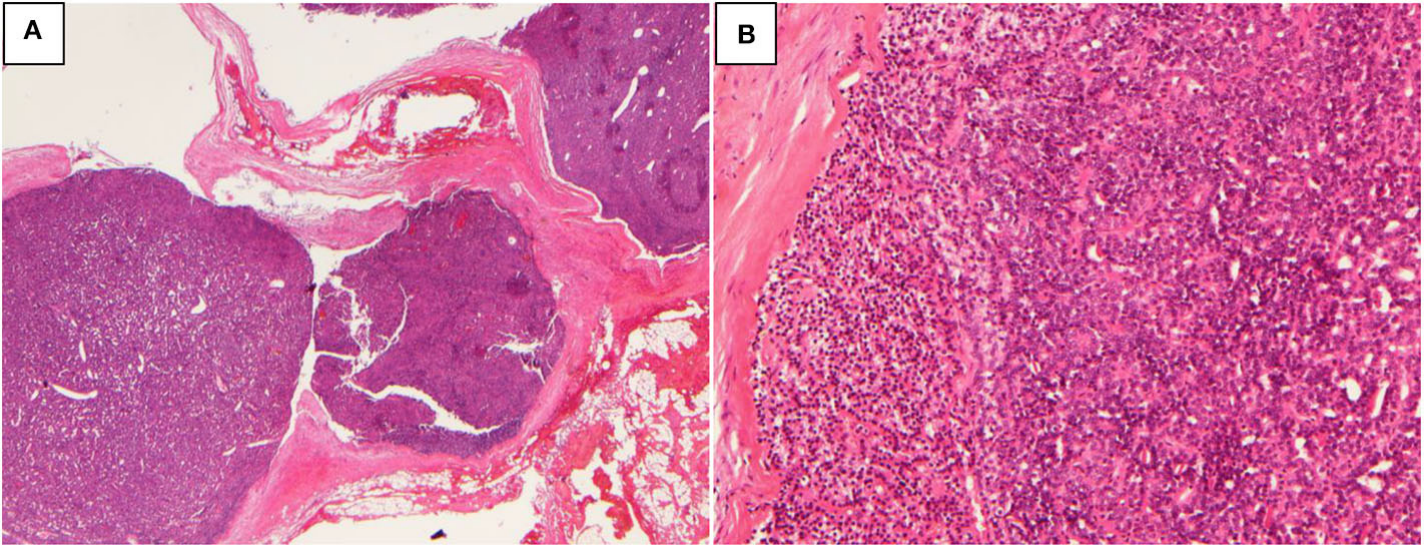
Atypical parathyroid neoplasm (patient 1). **(A)** 10× magnification, the tumor grows as multiple nodules with fibrous bands; **(B)** 100× magnification, the tumor cells are arranged in sheets or glands.

A 51-year-old woman presented with recurrent nephrolithiasis since 1992, along with bone pain-induced restricted movement since 2013. Her laboratory investigations revealed hypercalcemia (3.15 mmol/L, normal range: 2.13–2.70 mmol/L) and elevated serum PTH levels (2,136 pg/mL, normal range: 13–65 pg/mL). Ultrasonography detected bilateral renal calculi. Accordingly, in 2013, the patient underwent left superior parathyroidectomy for the pathology parathyroid adenoma in other hospital. Her postoperative serum Ca level was 1.96 mmol/L and PTH level was 22.8 pg/mL (decreased 98.9%). Until 2019, she experienced recurrent nephrolithiasis, with a serum Ca level of 2.56 mmol/L, iCa level of 1.31 mmol/L, and PTH level of 2189.4 pg/mL. Her BMD, as measured by DXA, was 1.232 in the lumbar spines 1–4 (T score 0.7), 0.887 in the femur neck (T score −0.2), and 0.823 in the total hip (T score −1.1). The parathyroid 99mTc-sestamibi scanning revealed multiple lesions. Meanwhile, 3 abnormal nodules (of size ~1.3 × 1.1 cm) were detected in the pancreatic tail through Gadolinium-enhanced abdominal MRI. Moreover, nodular masses were detected in both the adrenal glands. No significant abnormalities were recorded in the pituitary MRI. Genetic testing of *MEN1* revealed a heterozygous mutation: c.917T > G (p.L306R, exon 6). The patient had a family history of MEN1 (2 daughters and 1 grandson, 1 elder sister and her son, and 2 younger sisters and one of their sons were clinically diagnosed with PHPT, involving mutation at the same site in *MEN1*). Since the patient was diagnosed as recurrence of MEN-HPT, she underwent the second parathyroidectomy, whereby all of the remaining left inferior, right inferior, and superior parathyroid glands were removed and the left inferior parathyroid tissue (measuring 0.3 × 0.3 × 0.4 cm) were auto-planted into the right sternocleidomastoid muscle. The pathology was found to be atypical parathyroid neoplasm of the right superior and right inferior glands (Figure 1), along with the adenoma of the left inferior gland. Her postoperative serum Ca and PTH (decreased 97.1%) levels reverted to the normal since the first day after surgery, without any complications. At 1-year follow-up, the patient showed no signs of recurrence or metastasis, and no new disease associated with MEN1 appeared.

#### Case 2

A 52-year-old woman presented with a 10-year history of recurrent nephrolithiasis since 1995. In 2005, her laboratory investigations detected hypercalcemia (3.35 mmol/L), hypercalciuria (22.56 mmol/d), and elevated serum PTH levels (561 pg/mL). Her ultrasound detected bilateral renal calculi. She reported no bone pain or any history of fracture. BMD, as measured by DXA, was 0.998 in lumbar spines 1–4 (T score −0.9), 1.027 in the femur neck (T score 0.9), and 0.993 in the total hip (T score 0.3). At the age of 51 years, the patient was diagnosed with macroprolactinomas and the chiasmal compression and visual impairment occurred in a short period of time, the patient required pituitary adenoma resection by a single nostril transsphenoidal approach. The patient had no family history of MEN1, nephrolithiasis, or fractures. The genetic testing of *MEN1* revealed a heterozygous mutation: c.431T>C (p.F144S, exon 2). The patient, accordingly, underwent parathyroidectomy for the removal of her left superior and right inferior parathyroid glands—the pathology being atypical parathyroid neoplasm. Her postoperative serum Ca and PTH (decreased 87.5%) levels reverted to the normal since the first day after surgery, without any complications. At her 3-year follow-up, the patient showed no signs of recurrence or metastasis, and no new disease associated with MEN1 appeared. However, the patient has been lost to follow-up since 2008.

#### Case 3

A 49-year-old man presented with a 12-year history of recurrent nephrolithiasis since 2004. In 2015, his laboratory investigations revealed hypercalcemia (2.83 mmol/L) and elevated serum PTH levels (380 pg/mL). His ultrasonography detected bilateral renal calculi. His BMD measurement findings by DXA were 1.005 in lumbar spines 1–4 (T score −0.2), 0.83 in the femur neck (T score −0.5), and 0.815 in the total hip (T score −1.2). During the disease course, the patient often suffered from acid reflux and epigastric pain, occasionally accompanied with melena. The contrast-enhanced computed tomography (CT) of his abdomen revealed a highly-vascular lesion in the pancreatic head. The patient was accordingly diagnosed with pancreatic neuroendocrine tumors, for which he underwent ^125^I particle implantation and pancreatic biopsy—the pathology being pancreatic neuroendocrine tumors. Meanwhile, a subcutaneous mass of abdominal wall and mediastinum lesion (as per chest CT imaging of a 2.6 × 3.4-cm mass in the right mediastinum) were detected during his regular medical examinations. The patient underwent mediastinal mass resection and abdominal mass resection—the pathology being atypical carcinoid (mediastinum) and lipoma (abdominal wall). The patient had a family history of gastrointestinal diseases: his father died of gastric perforation at the age of 65 years, while his elder brother died of stomach cancer. Genetic testing of *MEN1* revealed a heterozygous mutation: c.549G>C (p.W183C, Exon 3). After clinical and genetic diagnosis as MEN1, the patient underwent left inferior and right superior parathyroidectomy—the pathology being carcinoma. His minimum postoperative serum Ca level was 2.39 mmol/L, while the minimum PTH level was 89 pg/mL (decreased 76.6%). After a year of surgery, his serum Ca level increased to 2.93 mmol/L, while the PTH level was 134 pg/mL. Although the parathyroid 99mTc-sestamibi scanning identified a new lesion in the left superior gland, the patient refused to undergo a second surgery. During the 4-year follow-up, the patient experienced only occasional diarrhea, and his serum Ca level ranged between 2.8 and 3.0 mmol/L, while his PTH level ranged between 120 and 150 pg/mL. No new disease associated with MEN1 appeared during follow-up period.

### Comparison of the PC/APN Group vs. the Non-PC/APN Group

A total of 18 MEN1-PC cases and 1 MEN1-APN case have been reported previously (3, 5, 6, 11–21). Table 4 summarizes the general characteristics, biochemical marker, treatments, histopathological features, and *MEN1* genetic testing of the PC/APN group (*n* = 21). Multiple types of gene mutations were detected in the MEN1-PC/APN cases, mainly belonging to missense mutations, followed by nonsense mutations, deletion mutations, insertion mutations, and duplicated mutations. The mean disease onset age of the PC/APN-group patients was 49.7 ± 10.4 years, and the group included 10 women and 11 men. In this group, the mean serum Ca level was 3.15 ± 0.44 mmol/L, and the median PTH level was 327 pg/mL (214.1, 673.1). Both the serum Ca and PTH levels were significantly higher in the PC/APN group than in the non-PC/APN group (Ca: 2.78 mmol/L [2.61, 2.88], PTH: 185.5 pg/mL [108.3, 297.0]; *P* = 0.0003, 0.0034, respectively), while the mean disease onset age was similar between the two groups (*P* = 0.065).

Table 4MEN1-PC/APN reported in the literature and the present study (3, 5, 6, 11–21).

**Table:** 

**A**							
**Case**	**Author**	**Publication year**	**Sex**	**Age at diagnosis (years)**	**Other MEN1 diseases**	**Ca (mmol/L)**	**PTH (Normal value)**
1	Wu et al. (11)	1992	M	48	PIT	3.15–4.05	1.47 nmol/L (0.10–0.46)
2	Sato et al. (12)	2000	F	51	N	2.67	/
3	Dionisi et al. (13)	2002	M	35	PET SL	3.35	75.01 pmol/L (1.1–5.83)
4	Agha et al. (14)	2007	F	69	PIT PET	3.9	37.7 pmol/L (0.9–5.4)
5	Agha et al. (14)	2007	M	32	PET	3.7	28 pmol/L (0.9–5.4)
6	Shih et al. (15)	2009	F	53	PIT PET AA	3.35	143.66 pmol/L (1.59–6.89)
7	Kalavalapalli et al. (16)	2010	F	40	PIT PET	2.65	7.2 pmol/L (1.59–6.89)
8	Del Pozo et al. (17)	2011	M	44	PET AA	3.1	21.83 pmol/L (0.75–5.67)
9	Joudele et al. (18)	2011	F	39	PIT PET AA SL	3.35	34.3 pmol/L (1.18–8.43)
10	Lee et al. (19)	2013	F	59	PIT AA	3.18	26.31 pmol/L (1.38–5.73)
11	Singh Ospina et al. (5)	2016	M	62	PET AA CT	3.1	14 pmol/L (1–5.2)
12	Christakis et al. (6)	2016	M	54	PET CT	2.63	42 pg/mL (9–80)
13	Christakis et al. (6)	2016	M	55	PIT PET AA	3.45	673.1 pg/mL (9–80)
14^*^	Christakis et al. (6)	2016	F	32	PET CT	3	/
15	Omi et al. (20)	2018	M	40	PIT PET	2.7	203 pg/mL (16–65)
16	Cinque et al. (3)	2017	M	61	PET AA	/	/
17	Cinque et al.	2017	M	55	AA	iCa 1.48 (1.12–1.32)	286 pg/mL (10–65)
18	Di Meo et al.	2018	M	62	PET	2.92	391.7 pg/mL
19^*^	Present study		F	51	PET AA	2.56	2189.4 pg/mL
20^*^	Present study		F	52	PIT	3.35	708.8 pg/mL
21	Present study		M	51	PIT PET AA	2.83	380 pg/mL

**Table:** 

**B**				
**Case**	**Surgery**	**Histopathologic features**	**Recurrence/metastasis**	***MEN1*** **mutation**
1	Single gland parathyroidectomy	Nests of uniform cells separated by dense collagenous septa and mitotic figures	Neck and sternum metastasis	/
2	TPTX + autoplantation	Mitoses in parenchymal cells, nuclear polymorphism and capsular invasion	N	c.842delC
3	First surgery: single gland parathyroidectomy Second surgery: SPTX	Uniform cytology, karyokinesis, fibrous bands, extracapsular, and vascular invasion.	Mediastinal metastasis	/
4	Resection of 3 parathyroid glands	Dense fibrous capsule, infiltrative cells, mitoses, desmoplasia, fat necrosis, and fibrous banding	Mediastinal metastasis	/
5	Resection of 2 parathyroid glands	Multiple mitoses with atypical features, fibrous banding, and thick fibrous capsule.	N	/
6	Surgical resection of 5 benign parathyroid glands plus a combined thyroid/parathyroid mass, followed by radiation therapy	Wide fibrous bands with vascular and perineural invasion and tumor fat necrosis. Focal invasion	N	c.1406_13dup8
7	First surgery: single gland parathyroidectomy Second surgery: SPTX	Very cellular parathyroid gland.	Lung metastasis.	/
8	Resection of 3 parathyroids + Hemithyroidectomy	Dense solid areas are consisting of oncocytic cells, desmoplastic stroma, focal necrosis and atypical cytology. Infiltration of the thyroid gland	Infiltration of the thyroid gland	c.549G>C (p.W183C)
9	Resection of 2 parathyroids + Hemithyroidectomy + Neck lymphadenectomy	Capsular invasion	Infiltration of the thyroid gland	c.129insA
10	Single gland parathyroidectomy + Hemithyroidectomy	Abnormal morphology within fibrous connective tissue and capsular invasion	Infiltration of the thyroid gland	/
11	First surgery: single gland parathyroidectomy Second surgery: single gland parathyroidectomy Third surgery: single gland parathyroidectomy	Histological evidence of local invasion (nerve), fibrous bands and nuclear atypia	Esophageal recurrence	/
12	First surgery: SPTX Second surgery: TPTX	Lobular features, mitoses, and capsular invasion	Recurrence	c.703G>A (p.E235K)
13	SPTX	Capsular invasion, fibrosis, nuclear polymorphism	Recurrence	c.1378C>T (p.R460X)
14^*^	First surgery: resection of 2.5 parathyroids Second surgery: single gland parathyroidectomy	Lobular features and fibrous bands	N	/
15	First surgery: resection of 3 parathyroids + Hemithyroidectomy + autoplantation Second surgery: Completion thyroidectomy + resection of 1 intrathyroidal mass	Capsular invasion and vascular invasion	Infiltration of the thyroid gland	/
16	Resection of 2 parathyroids	Capsular invasion	Infiltration of esophageal	c.1252G>A (p.D418N)
17	Single gland parathyroidectomy	Capsular invasion	Infiltration of surrounding adipose tissue	c.1252G>A (p.D418N)
18	Resection of 2 parathyroids + Hemithyroidectomy + Neck lymphadenectomy	/	N	/
19^*^	First surgery: single gland parathyroidectomy, Second surgery: TPTX + autoplantation	Lobular features, fibrous bands, focal active growth, and mitoses	N	c.917T>G (p.L306R)
20^*^	Resection of 2 parathyroids	Active growth, mitoses, and thick bands	N	c.431T>C (p.F144S)
21	Resection of 2 parathyroids	The cubical or rounded cancer cells are small, stratified in layers as a ridge, focal thick bands, capsular invasion, suspicious vascular invasion, focal oncocytic cells	Recurrence	c.549G>C (p.W183C)

Note: 1. *: MEN1-APN cases. *2. PIT, pituitary adenoma; PET, pancreatic endocrine tumor; AA, adrenal adenoma; CT, carcinoid tumor; SL, subcutaneous lipoma; TPTX, total parathyroidectomy; SPTX, subtotal parathyroidectomy; N, no; /, unknown; Ca, serum calcium; PTH, serum parathyroid hormone*.

## Discussion

PC is a rare endocrine malignant tumor, with an estimated prevalence rate of 0.005% of all reported cancer cases (22). Until date, <2,000 cases of PC have been reported in the literature (22), which accounts for <1% of all cases of PHPT in the Western countries and among the Caucasian population (23). However, in the Chinese population, carcinoma accounts for 5–7% of PHPT cases (24, 25). The occurrence of parathyroid malignant tendency tumors (PC/APN) in MEN1 is a rare phenomenon, and its prevalence has so far been reported in only 2 centers in the USA. Of the 348 MEN1 patients reported by Mayo Medical Center, only 1 (0.28%) had a pathological diagnosis of PC (5). In addition, of the 291 MEN1 patients reported by the MD Anderson Cancer Research Center, only 3 (1%) had parathyroid malignant-tendency tumors (0.7% for PC and 0.3% for APN) (6). In this study, of the 153 MEN1-HPT patients admitted to our center, 2 had APN (1.3%) and 1 had PC (0.7%), with a total prevalence rate of 2.0%. The proportion of both sporadic PC and MEN-PC/APN is relatively high in the Chinese population, probably due to factors such as race and lifestyle (25).

As such, the total number of cases of MEN1-PC/APN patients in the literature and at our center has come to 21. Twelve of these patients have serum Ca level >3.0 mmol/L (Table 4) and PTH level about 5-times of the upper limit of the reference range. As compared with that in the non-PC/APN group at our center, the serum Ca and PTH levels of the MEN1 patients were significantly higher in the MEN1-PC/APN group. Singh et al. reported the clinical data of 11 MEN1-PC patients and compared them with those of 510 PC cases unrelated to MEN1 (5). They found no significant differences with respect to the gender, age, and PTH levels between the two groups (5, 26, 27). These results suggest that the clinical features of MEN1-PC/APN are more similar to those of sporadic PC than to those of MEN1-benign parathyroid tumors. Therefore, MEN1 patients showing a high level of serum Ca and PTH levels should be considered as potential PC/APN cases.

The MEN1-PC/APN patients in the current study presented with a near-normal BMD, which is different from the previous study (20, 21). Because of the small sample size, it could only reflect the individual's condition and might not be consistent with the typical characteristics of MEN1-PC/APN. Eller-Vainicher et al. have reported that MEN1-related PHPT (MHPT) patients showed lower BMD of the lumbar spine and femoral neck than sporadic PHPT (SHPT) patients, even though MHPT patients exhibited milder biochemical presentation than their SHPT counterparts (28). Therefore, increased severity of bone loss in MHPT patients could be partially attributed to the combination of other endocrine dysfunction components, including hypercortisolism, hyperprolactinemia, hypogonadism, and GH deficiency, which also exert negative effects on bone mineralization (29). However, in the previous study of our center, there were no differences between MHPT and SHPT were observed in bone indices as measured using both DXA and high-resolution peripheral computed tomography (HR-pQCT) (4, 30). Therefore, more research is needed in the future to clarify the bone changes in MHPT patients, especially MEN1-PC/APN patients.

Eleven of the 21 MEN1-PC/APN cases carried 9 *MEN1* mutations, 2 of which belonged to the same *MEN1* family (c.1252 G> A, p.D418 N, exon 9). In this study, the mutation of the PC patient was the same as that in a previously reported PC patient (c.549 G> C, p.W183C, exon 3) (19). Two *MEN1* mutations were identified in APN patients (c.917 T> G and c.431T> C). The cases of MEN1-PC/APN were too limited to analyses for the genotype–phenotype relationship, necessitating the analysis of more number of cases for an elaborate study in the future.

The diagnosis of PC and APN depends on the histopathological features, which were relatively complicated and challenging to decipher (21, 31). Presently, the morphological characteristics of PC mainly includes fibrous bands arranged in a trabecular design, mitotic activity, enlarged nucleoli, and capsular and vascular invasion (32). Among all these features, capsular and vascular invasion is considered the most relevant one to indicate tumor recurrence and distant metastasis, and is hence the most reliable evidence for the diagnosis of malignant tumors (33). Moreover, it is believed that immunohistochemical analysis can improve the diagnostic accuracy of PC. The loss of expression of parafibromin combined with those of protein gene product 9.5 (PGP 9. 5) and galectin-3 have also been reported to be common in PC. Moreover, Ki-67 index is usually >5% in malignant tumors. The histopathological characteristics of APN, as a particular pathological type between adenoma and carcinoma, are also not clearly defined. It is generally believed that APN can be diagnosed based on the presentation of some atypical features of carcinoma, except for the lack of the invasion to the neighboring structures (6). Neither of the 2 MEN1-APN patients at our center reported metastases or adhesion with the adjacent tissues, and their pathological findings revealed active growth, mitoses, and thick fibrous bands, but no capsular or vascular invasion. For these 2 patients who were diagnosed with APN, longer term follow-up is required to clarify the biological behavior of their pathology. Interestingly, in case PC is pre-operatively suspected, to prevent the risk of needle tract implantation metastases and the tissue diffusion caused by capsule rupture, fine needle aspiration should be avoided (34, 35).

The surgical removal of abnormally overactive parathyroid glands in MEN1-HPT patients is a definitive treatment (2). As quite a few MEN1-HPT cases involved multiple glands and were susceptible to relapse, the Clinical Practice Guidelines for MEN1 recommend subtotal parathyroidectomy (SPTX, removing at least 3.5 glands) as the first choice of treatment (2). Total parathyroidectomy (TPTX) with auto-plantation can be considered in case of extensive parathyroid lesions. The incidence of hypoparathyroidism after the local resection of parathyroid was reduced in a previous study; however, the recurrence rate was 3.11-times more than that of SPTX or TPTX (36). There is no guideline available for MEN1-PC/APN, considering its relatively rare nature. All the 21 cases of MEN1-PC/APN so far have undergone 1–3 surgeries, and 1 of these even received postoperative radiation therapy for thyroid invasion. Only 1 patient undertook TPTX as the first operation and 2 patients as the second operation, and, in both the situations, no recurrence or metastasis was recorded after TPTX. Seven patients underwent a single gland parathyroidectomy as the first operation, 5 of whom had metastases (of the neck and sternum, mediastinum, lung, thyroid, and esophagus), and 1 of them had a recurrence. All of the metastases/recurrence patients had a second operation, except for 1 who had no report of a subsequent treatment. It is important for the surgeon to be aware of the appearance of PC during surgery. However, PC/APN is often not conclusively identified even during the operation. Based on the accumulated experiences, if MEN1-PC/APN is pre-operatively suspected, it is recommended that a surgical approach be selected in accordance with the nature of PC. Clayman et al. and Shane et al. suggested the following surgery plan: (1) Completely remove the lesion and its adhesion tissues (including the anterior cervical muscle group, esophageal muscle group, and recurrent laryngeal nerve) to avoid tumor rupture and overflowing; (2) also remove the ipsilateral thyroid lobe, paratracheal lymph node, and superior mediastinal lymph node; and (3) if the evidence suggests the involvement of central lymph nodes, the VI level of cervical lymphadenectomy should be performed (37, 38).

More importantly, the management of postoperative patients remains challenging. Both, the recurrence of PC/APN and the proliferative growth of the remaining glands can cause postoperative hypercalcemia. Therefore, if postoperative hypercalcemia is identified, the reason for its occurrence should be clarified immediately. Due to the proliferative growth of the remaining glands, it is suggested that only limited resections be performed via minimally invasive surgery (6). However, if there is sufficient evidence supporting possible PC/APN recurrence in the surrounding tissues, the approach should be open and involving adequate exposure (6).

The authors acknowledge that this study has some limitations. For instance, this being a retrospective study conducted in a single center, there is a possibility of information and confounding biases attributable to the small sample size and overlooked/missed data. However, considering the rarity of MEN1-PC/APN, even a small cohort has the potential to provide valuable data and experience to clinicians.

In summary, MEN1-PC/APN is a relatively rare disease, with an overall prevalence rate of only 2.0% in a Chinese MEN1-HPT cohort. This rate is slightly greater in Chinese population than that reported for Caucasian population. The serum Ca and PTH levels in patients with MEN1-PC/APN are usually greater than in those with MEN1-associated benign parathyroid tumors. However, MEN1-PC/APN is often not conclusively identified pre-operatively and the diagnosis based upon pathology most of the times. If this disease is pre-operatively suspected, surgical treatment is best recommended following PC.

## Data Availability Statement

All datasets presented in this study are included in the article/supplementary material.

## Ethics Statement

The studies involving human participants were reviewed and approved by the Ethics Committee of PUMCH. Written informed consent was obtained from the individuals for the publication of any potentially identifiable images or data included in this article (No patient was under the age of 16, or otherwise legally or medically unable to provide written informed consent).

## Author Contributions

AS, YY, MN, and OW are involved in experimental and analysis. AS, OW, and XX are involved in analysis. SL is involved in conception, analysis, and writing. All authors contributed to the article and approved the submitted version.

## Conflict of Interest

The authors declare that the research was conducted in the absence of any commercial or financial relationships that could be construed as a potential conflict of interest.
